# Type 2 diabetes outcomes in patients with Posttraumatic stress disorder and psychiatric comorbidity

**DOI:** 10.1016/j.diabres.2026.113257

**Published:** 2026-04-14

**Authors:** Ana I. Maldonado, Joanne Salas, Paula P. Schnurr, Beth E. Cohen, Allan S. Jaffe, Kenneth E. Freedland, Patrick J. Lustman, Matthew Friedman, Jeffrey F. Scherrer

**Affiliations:** a VA Palo Alto Healthcare System, Palo Alto, CA, United States; b VA Palo Alto Cooperative Studies Program Coordinating Center, Palo Alto, CA, United States; c The Advanced HEAlth Data (AHEAD) Research Institute, Saint Louis University School of Medicine, St. Louis, MO, United States; d Department of Health and Clinical Outcomes Research, Saint Louis University School of Medicine, St. Louis, MO, United States; e National Center for PTSD, White River Junction, VT, and Department of Psychiatry, Geisel School of Medicine at Dartmouth, Hanover, NH, United States; f Department of Medicine, University of California San Francisco School of Medicine and San Francisco VAMC, San Francisco, CA, United States; g Department of Cardiovascular Medicine and Department of Laboratory Medicine and Pathology. Mayo Clinic, Rochester, MN, United States; h Department of Psychiatry, Washington University School of Medicine, St. Louis, MO, United States; i Department of Family and Community Medicine, Saint Louis University School of Medicine. 1402 South Grand Blvd, St. Louis, MO, United States; j Department of Psychiatry and Behavioral Neuroscience, School of Medicine, Saint Louis University, St. Louis, MO, United States

**Keywords:** Epidemiology, Posttraumatic stress disorder, Veterans, Type 2 diabetes, Observational, Cohort

## Abstract

**Aims:**

Posttraumatic stress disorder (PTSD) is associated with type 2 diabetes (T2D). However, no studies have determined if risk for adverse T2D outcomes vary among different PTSD comorbidity patterns.

**Methods:**

We identified 152,171 Veterans Health Administration (VHA) patients with de-identified medical records who had comorbid PTSD and T2D. All had adequate HbA1c control, not on insulin, and were free of microvascular and macrovascular complications at index. Latent class analyses (LCA) identified 3 comorbidity profiles characterized as depression + anxiety; high comorbidity; and low comorbidity. We then estimated the link between comorbidity classes and risk for adverse T2D outcomes and all-cause mortality.

**Results:**

Most (64.4%) of the sample was ≥ 60 years of age, male (90.9%), and White race (69.1%). High co-morbidity vs. low comorbidity and depression + anxiety vs. low comorbidity were associated with all-cause mortality (HR = 1.39; 95%CI:1.28–1.50 and HR = 1.18;95%CI:1.14–1.23, respectively). The depression + anxiety vs. the low comorbidity class, had slightly worse glycemic control (HR = 1.04; 95%CI:1.02–1.06) and microvascular complications (HR = 1.04; 95%CI:1.02–1.06).

**Conclusions:**

More psychiatric comorbidity in patients with PTSD is associated with worse glycemic control, increased risk for T2D micro- and macro- complications, and mortality. Research is needed to determine if treatment and improvement in patients with comorbid psychiatric disorders leads to improved T2D outcomes.

## 1. Introduction

Posttraumatic stress disorder (PTSD) is a modifiable risk factor for type 2 diabetes (T2D) [1]. There is emerging evidence that PTSD also impacts T2D outcomes [2,3]. Encouragingly, one study on a large cohort of individuals with comorbid PTSD and T2D found that large improvements in PTSD symptoms, even after controlling for comorbid mood disorders and substance use disorders, were associated with modest reduction in risk for starting insulin, microvascular complications and all-cause mortality [2]. These results were based on models that controlled for comorbid psychiatric conditions, which are highly prevalent among those with PTSD [4–6]. In these studies, psychiatric comorbidities were conceptualized as potential confounders of the relationship between PTSD and T2D, rather than as potential predictors. Consequently, little is known about how patterns of comorbid psychiatric disorders affect T2D outcomes in persons with PTSD.

Individuals with PTSD rarely present with a single psychiatric diagnosis; rather, they often suffer from a constellation of co-occurring mental health conditions [4–6]. Previous research has predominantly adopted a methodological approach that isolates the association between PTSD and health outcomes, treating other psychiatric conditions as confounding variables. By investigating the patterns of psychiatric comorbidity in PTSD, the present study better reflects the T2D outcomes related to the clinical complexity of patients with PTSD.

To our knowledge, no research has determined how different PTSD comorbidity profiles relate to T2D outcomes. This is an important gap in knowledge, particularly within the Veterans Health Administration (VHA) where more than 70% of those with PTSD have at least one co-morbid psychiatric disorder [7]. Understanding which specific profiles of co-occurring psychiatric conditions confer the greatest risk for adverse diabetes outcomes is essential for developing targeted prevention and treatment strategies. To address this gap, we used a retrospective cohort of VHA patients with a PTSD diagnosis to develop latent comorbidity profiles. We then estimated the association between different PTSD-psychiatric comorbidity profiles and risk for poor T2D outcomes, including starting insulin, microvascular complications, glycemic control and all-cause mortality.

## 2. Methods

### 2.1. Subjects

De-identified VHA administrative medical record data from 10/01/2011 to 9/30/2022 (fiscal years (FY): FY12-FY22) was used to create a retrospective cohort and study variables. Medical record data include diagnostic codes (ICD-9 and ICD-10), procedural terminology (CPT) and ICD-9 and −10th revision of the Procedure Coding System (PCS) codes, medication fills, laboratory results, vital signs, vital status, and demographic information. Health care purchased outside of the VHA was included in our database.

Because the data were de-identified and the investigators could not re-identify patients, the Saint Louis University and VHA Institutional Review Boards deemed this study exempt. This study followed the Strengthening the Reporting of Observational Studies in Epidemiology (STROBE) guidelines.

### 2.2. Eligibility

VHA data managers maintained the sample on a secure VA server. VA released a base sample to investigators that included 2,111,947 patients with at least one PTSD diagnosis code present in FY12-FY22. We then applied a validated PTSD case finding algorithm, where confirmed PTSD was defined as the presence of at least two diagnostic codes (ICD-9: 309.81; ICD-10: F43.1*) on at least two separate outpatient visits within a 12-month period or one inpatient stay (n = 1,515,208). Confirmed PTSD date is either inpatient stay date or the second diagnostic code (for outpatient visits) meeting the algorithm. Using 2 diagnostic codes in 4 months has an 82% positive predictive value when compared to a gold standard PTSD Checklist (PCL) score ≥ 50 [8], and presence of 2 diagnostic codes has an 88.4% agreement with the Structured Clinical Interview for DSM-IV (SCID) lifetime PTSD diagnosis [9]. This is better or equal to World Health Organization Composite International Diagnostic Interview diagnoses compared to gold standard Structured Clinical Interview for DSM Disorders [10,11].

Eligibility criteria were applied in FY14-FY20 (n = 1,465,368 patients) to create a dynamic cohort, where patients entered the analytic cohort when they first met eligibility criteria below. Additionally, this allowed for a 2-year lookback and a maximum of 2 to 9 years of follow-up time.

Of those who met the PTSD criteria in FY14-FY20, we then selected patients with comorbid T2D in the year before PTSD diagnosis (n = 413,992). We excluded patients with insulin fills, type 1 diabetes and any microvascular complications in the 2-years prior to index date (n = 236,192 patients remaining). Among these patients, the index date was defined as the earliest date that patients met our PTSD case definition. Patients were then required to have controlled T2D at baseline, defined as the last HbA1c value in the year prior to index < 7.5%. Patients were 18–80 years old at index. After removing patients with missing demographic data, the final analytic sample contained 152,171 patients with comorbid PTSD and T2D who had adequate HbA1c control, were not on insulin and were free of microvascular complications at index date.

### 2.3. Variables

Detailed definitions of all study variables are shown in the Appendix e-Table 1.

### 2.4. Primary outcomes

We used multiple indicators of poor T2D outcomes including insulin initiation, poor glycemic control (HbA1c ≥ 7.5%), any microvascular complications (i.e., presence of any ICD-9/10 diagnostic codes for diabetic nephropathy, retinopathy, or neuropathy), and all-cause mortality.

Insulin has traditionally been used as augmentation therapy; therefore, we defined insulin initiation as a marker of poor T2D control. Recommendations on glycemic control vary and HbA1c goals are based on patients’ age, comorbidities, presence of microvascular complications, and treatment preferences. Tight control with a goal HbA1c < 7% is recommended by the American Diabetes Association and VHA-Department of Defense Clinical Practice Guideline for many non-pregnant adults. Alc goals are more flexible for patients with limited life expectancies and/or increased risk for hypoglycemic complications. Thus, we used a cut off of an HbA1c < 7.5% to define good control and considered HbA1c ≥ 7.5% during follow up as poor glycemic control [12,13].

The VHA’s vital master file includes dates of death gathered from VHA medical records, the Social Security Administration, the Beneficiary Identification Records Locator Subsystem, and Center for Medicare and Medicaid Services to measure all-cause mortality.

### 2.5. Secondary outcomes

Secondary outcomes included incident macrovascular complications, cardiovascular disease (CVD), and peripheral vascular disease (PVD), defined by the presence of at least one ICD-9 or −10 code for each disease in follow-up. Cardiovascular disease was a composite defined by ICD-9 or −10 codes for ischemic heart disease, cardiomyopathy, atrial fibrillation, and other arrhythmias. Secondary outcomes were examined among a subsample of patients free of CVD and PVD at baseline (i.e., absence of any ICD-9 or −10 code in the 2-years prior to baseline).

### 2.6. Exposure – psychiatric comorbidities

Eight psychiatric comorbidities were measured in the 2-years prior to index using ICD-9/10 diagnostic codes, and included depression, anxiety (i.e., a composite of panic disorder, social phobia, generalized anxiety disorder, obsessive compulsive disorder, and anxiety disorder not otherwise specified), severe mental illness (SMI) (i.e. a composite of schizophrenia and bipolar disorder), smoking or nicotine dependence (also measured with social health history data), alcohol abuse/dependence, opioid abuse/dependence, stimulant abuse/dependence, and cannabis abuse/dependence. To classify patients into mutually exclusive PTSD comorbidity profiles using the eight psychiatric indices above, latent class analysis (LCA) was utilized [14,15]. LCA was performed using MPlus v8.8 [16]. Models were fit in a stepwise fashion starting with one latent class. Best fit was assessed using model fit statistics (e.g., log-likelihood, AIC, BIC, sample-size adjusted BIC, entropy), the adjusted Lo-Mendell-Rubin likelihood ratio test (aLMR) comparing a solution for k vs. k-1 classes, parsimony and meaningful interpretability of classes [17]. As it is common for fit statistics to continually improve and for aLMR to continually be significant with additional classes with such a large sample size, a scree plot of sample-size adjusted BIC against the number of classes was examined to identify the inflection point where sample-size adjusted BIC value declined. See Appendix e-Table 10 for example MPlus syntax for conducting a latent class analysis.

### 2.7. Covariates

Demographic covariates included age at index (18–39, 40–49. 50–59, 60–80), sex (male vs. female), race (White, Black, Other), marital status (married vs. not married), VA only versus VA plus other health coverage, and U.S. census region. We control for region because clinical practice patterns vary across the U.S. within the VHA. To control for detection bias, the average number of outpatient healthcare clinic encounters per month was calculated per patient. The distribution of outpatient clinic encounters per month was then categorized into high healthcare utilizers (top 25th percentile) versus not high (bottom 75th percentile).

All other covariates were measured in the 2-years prior to index using ICD-9/10 diagnostic codes or medication fills, unless otherwise noted. T2D severity was controlled for using baseline HbA1c value (the last available value in the year before index). Additionally, sustained use of metformin, sulfonylurea, and other diabetes drugs (e.g., DPP-4, GLP-1, SGLT2, or TZD) in the 2-years prior to baseline was coded. Sustained use was defined as at least two fills in any 6-month period. Psychiatric treatments may reduce the effect of PTSD and psychiatric comorbidities on T2D outcomes; therefore, we controlled for minimally adequate PTSD treatment and antidepressant medication (ADM) treatment as well as atypical antipsychotic use. We used procedure codes to measure use of any type of psychotherapy and included any type of antidepressant from the VA pharmacy data. Minimally adequate PTSD treatment was defined as ≥ 9 sessions in any 15-week period. Adequate ADM treatment was defined as at least 12 continuous weeks of fills for antidepressant medications which is considered acute phase depression treatment. We also controlled for sustained use of atypical antipsychotics. We controlled for the following physical comorbidities: obesity (last BMI before index ≥ 30), hyperlipidemia, atrial fibrillation, angina, congestive heart failure, hypertension, left ventricular hypertrophy, myocardial infarction, peripheral vascular disease, and stroke.

### 2.8. Analytic approach

To control for confounding in outcome models, meaningful differences between PTSD-comorbidity profiles were removed using entropy balancing (e-balance) [18,19]. E-balance is a data pre-processing method that assigns weights to each patient so that certain balance constraints are met (e.g., means and variance or distributional proportions are equal across groups). A pseudo-population is created so that distributions of covariates between exposure groups are balanced (i.e., equal or near equal). When e-balance weights are used, analyses compare groups as if they had been randomized. In this investigation, outcome analyses estimate the ‘average treatment effect’ (ATE). E-balance weights were computed using the *WeightIt* package in Rv4.2.1 [20]. Balance was evaluated using the standardized mean difference percent (SMD%=SMD*100), where an SMD% < 10% indicates good balance (e.g. equal covariate distributions) [21]. See Appendix e-Table 10 for example R-Studio *WeightIt* syntax.

The main analyses were conducted using SAS v9.4 at a two-tailed alpha = 0.05 [22]. Bivariate associations of covariates and PTSD-comorbidity groups were assessed using chi-square tests (for categorical variables) and one-way ANOVA (for continuous variables). Maximum SMD% between all pairwise group comparisons assessed covariate balance before and after e-balance weighting. Competing risk models [23], which account for the competing risk of death, were used to estimate hazard ratios (HR) and 95% confidence intervals (CI) for the association of PTSD comorbidity group and time to insulin initiation, time to poor glycemic control, time to any microvascular complication, and time to each macrovascular complication (i.e. CVD, PVD). Rather than treat death as a censored event, competing risk models treat death as an event that prevents the primary outcome of interest, which means patients would no longer be at risk. Time was measured as months from index to outcome, death or censor date. Censor date for insulin initiation, any microvascular outcome, CVD or PVD, in the absence of mortality, was last visit date in follow-up, while for poor glycemic control, censor date was last HbA1c measurement. Cox proportional hazard models estimated the association of PTSD comorbidity group and all-cause mortality, where censor date was the end of follow-up (9/30/22). All models were computed before and after e-balance weighting, and e-balance weighted models used robust, sandwich-type variance estimators [21]. The proportional hazard assumption was met for all models (p > 0.10).

Subgroup analyses for primary outcomes for each e-balance weighted model were computed for age, sex, and race. E-balance weighting constraints were created so that covariates balanced within each subgroup. An interaction term of each subgroup variable with PTSD comorbidity group assessed whether significant effect modification was present (p < 0.05).

Last, we conducted sensitivity analyses changing the definition of poor glycemic control as ≥ 8.0%.

## 3. Results

Most (64.4%) of the sample was ≥ 60 years of age, male (90.9%), and identified as White race (69.1%) (Table 1). The mean HbA1c at index was 6.3%(±0.6%) and nearly 38.8% were prescribed metformin. The most prevalent physical conditions were obesity (62.8%), hyperlipidemia (79.0%) and hypertension (79.7%). The most prevalent psychiatric diagnoses that were used to compute PTSD comorbidity profiles were depression (50.3%) and anxiety disorders (23.3%). The most prevalent substance use disorders were smoking (46.3%) and alcohol abuse/dependence (21.4%). Only 6.1% received minimally adequate duration of PTSD psychotherapy while 65.4% had adequate ADM treatment.

The best fitting LCA model was a 3-class solution based on parameters shown in Appendix e-Table 2. While fit indices kept improving and aLMR tests were significant with each additional class, a scree plot showing sample-size adjusted BIC against number of classes showed that BIC leveled off starting at 3-classes. (see Appendix e-Fig. 1). Additionally, for the four and five class solutions, one or more of the logit thresholds approached extreme values, meaning there was some degree of model non-identification and model parameters were unstable/unreliable. For the final 3-class solution, Class 1 (20.9% of the sample) was characterized by high probability of depression + anxiety, Class 2 was least common (7.1%) and was characterized by high comorbidity (i.e. elevated probability of having depression, anxiety and multiple type of substance use disorders) and Class 3 was most common (72.0%) and demonstrated low comorbidity for all diagnoses used in the LCA (Fig. 1). Item response probabilities given class membership and average latent class posterior probabilities by latent class are shown in Appendix e-Table 3 and Appendix e-Table 4, respectively. Henceforth, we refer to latent classes as ‘depression + anxiety’; ‘high comorbidity’; and ‘low comorbidity’.

As shown in Table 2, the SMD% revealed that 50–59 year olds were more likely to be in the high comorbidity group compared to the depression + anxiety or low comorbidity class (SMD%=44.9). Patients ≥ 60 years old were most common in the low comorbidity and depression-anxiety classes (SMD%=44.6). Black race was over-represented (SMD%=61.1) and white race was disproportionately under-represented in the high comorbidity class (SMD%=57.8). Patients with VA insurance only (SMD%=53.8) and high healthcare utilizers were more common in the high comorbidity group (SMD%=86.5). Obesity (SMD%=52.2) and hyperlipidemia (SMD%=70.4) were less common in the high comorbidity group. Receipt of adequate ADM treatment (SMD%=42.3) was least prevalent in the low comorbidity class and atypical antipsychotics were more prevalent in the high co-morbidity class (SMD%=60.6). After applying e-balance weights, there was no meaningful difference in the distribution of all covariates by latent class as indicated by all weighted SMD%<10.

For the overall sample during the 2–9 year follow-up, 11.5% started insulin, 38.9% had poor glycemic control, 23.9% had diabetic nephropathy, 33.7% neuropathy, 15.8% retinopathy, 40.8% had any microvascular complication, and 13.5% died. Table 3 shows the incidence rate per 1000 person years (PY) for each outcome by latent class. The incidence of starting insulin was greatest among the high comorbidity class, followed by the depression + anxiety class and was lowest in the low comorbidity class (p < 0.0001). The incidence of poor glycemic control was greatest in the low comorbidity class (99.9/1000 PY), intermediate in the depression + anxiety and lowest in the high co-morbidity class (p < 0.0001). Incident microvascular complications were significantly associated with latent class membership with the highest incidence among the low comorbid group, followed by the depression + anxiety group and lowest in the high comorbidity class (p < 0.0001). Mortality incidence was greatest among the high comorbidity class (p < 0.0001), intermediate in the depression + anxiety class and lowest in the low comorbidity class. Cumulative incidence functions, after entropy balance weighting, for non-mortality primary outcomes are available in Appendix e-Figs. 2 to 4.

Results from competing risk models and Cox proportional hazard models are shown in Table 4. After controlling for confounding with e-balance, we observed that compared to patients in the low comorbidity class, those in the depression + anxiety class and those in the high co-morbidity class, had a significantly greater risk for starting insulin and all-cause mortality. There were statistically significant but small hazard ratios for the association between the depression + anxiety, compared to the low comorbidity class and for the risks for poor glycemic control (HR = 1.04; 95%CI:1.02–1.06) and microvascular complications (HR = 1.04; 95%CI:1.02–1.06). High comorbidity vs. low comorbidity was not associated with differential risks for these two outcomes. Cox models revealed that high comorbidity vs. low comorbidity and depression + anxiety vs. low comorbidity were significantly associated with all-cause mortality (HR = 1.39; 95%CI:1.28–1.50 and HR = 1.18;95% CI:1.14–1.23, respectively).

Age subgroup analyses are shown in Appendix e-Table 5. There was significant effect modification by age for all outcomes. Except for those 18–39 years of age, all older age groups in the depression + anxiety class vs. the low comorbidity class, had significantly higher risk for starting insulin. In age groups 50–59 and 60–80, the risk of starting insulin in the high comorbidity group was greater than the depression + anxiety group as compared to the low comorbidity group. Additionally, patients 40–49 with high comorbidity vs. low comorbidity, were 22% less likely to develop poor glycemic control (HR = 0.88; 95%CI:0.81–0.96) and those 60 to 80 with depression + anxiety vs. low comorbidity had a 6% increased risk for poor glycemic control (HR = 1.06; 95%CI:1.03–1.09). For microvascular complications, patients 40–49 years of age in the depression + anxiety class (HR = 1.11; 95%CI:1.14; 95%CI:1.03–1.26) and in the high comorbidity class as compared to the low comorbidity class (HR = 1.14; 95%CI: 1.03–1.26) had significantly elevated risk for any microvascular complication. There was a small increased risk for microvascular complications in the depression + anxiety class vs. low comorbidity in those 50–59 and those 60–80. High comorbidity was not significantly associated with microvascular complications in these two age groups. All age groups had an elevated risk for all-cause mortality if they were in the depression + anxiety or the high comorbidity class as compared to the low comorbidity class. The magnitude of risk was greater in younger age cohorts with a significant age by class interaction (p < 0.0001).

Results from subgroup analyses by biological sex are shown in e-table 6. We observed a significant sex by class interaction (p = 0.0002) in risk of starting insulin. No other significant sex by class interactions were found for other T2D outcomes. The depression + anxiety class compared to the low comorbidity class was significantly associated with increased risk for starting insulin for both men and women. Only among men was the high comorbidity group significantly associated with increased risk for insulin initiation (HR = 1.32; 95%CI:1.25–1.39).

Subgroup comparisons by race are shown in e-table 7. There was significant effect modification by race for time to poor glycemic control (p = 0.002) and microvascular complications (0.0003), but not mortality or starting insulin. Among White and Black, but not Other race, there was a 3–6% increased risk of poor glycemic control comparing depression + anxiety vs. low comorbidity. High comorbidity vs. low comorbidity was associated with a 21% increased risk (HR = 1.21; 95% CI: 1.05–1.41) of poor glycemic control in “Other” race, but not associated with poor glycemic control in White or Black patients. For microvascular complications, among White and Black race, depression + anxiety vs. low comorbidity was associated with a 4% increased risk of microvascular complications. High vs. low comorbidity was not associated with microvascular complications for Black or White patients, but associated with a 31% lower risk among “Other” race patients (HR = 0.69; 95%CI: 0.58–0.82).

Results of analyses for secondary, macrovascular outcomes are shown in Appendix e-Table 8 and e-Table 9. Among the entire sample of patients, (n = 152,171), 48,911 patients with CVD prior to baseline and 8,022 with prior PVD (not mutually exclusive) were excluded from analyses, leaving 100,610 patients free of CVD and PVD at baseline. Overall, 33.8% of patients (incidence rate = 71.4/1,000 PY) developed CVD in follow-up and 13.1% (incidence rate = 23.7/1,000 PY) developed PVD in follow-up. For CVD, the incidence rate was lowest among low comorbidity (69.6/1,000 PY), increased to 75.0/1,000 PY among depression + anxiety, and was highest among patients with high comorbidity (81.4/1,000 PY). Rates of PVD did not significantly differ between comorbidity groups. After controlling for confounding using e-balance, it was found that compared to low comorbidity, depression + anxiety (HR = 1.11; 95%CI = 1.08–1.14) and high co-morbidity (HR = 1.13; 95%CI = 1.09–1.18) had an 11–13% increased risk of CVD. Similarly, after e-balance weighting to control for confounding, depression + anxiety (HR = 1.10; 95%CI = 1.05–1.14) and high comorbidity (HR = 1.11; 95%CI = 1.04–1.18) had a 10–11% increased risk of PVD.

Sensitivity analyses using HbA1c ≥ 8.0% as an indicator of poor glycemic control, 31.4% of the overall sample and 29.6% of depression + anxiety, 28.3% of high comorbidity, and 32.2% of low comorbidity met the threshold for poor glycemic control. After e-balance weighting, results were similar to the original primary outcome definition of HbA1c ≥ 7.5%. Compared to low comorbidity, depression + anxiety was associated with a 4% increased risk of poor control (HR = 1.04; 95%CI = 1.02–1.06) and high comorbidity was unrelated to poor control (HR = 1.01; 95%CI = 0.97–1.04).

## 4. Discussion

In this retrospective cohort study of U.S. veterans with co-morbid PTSD and T2D receiving care at the VHA, we identified distinct latent classes of psychiatric comorbidities and examined their associations with adverse T2D outcomes over a 2 to 9 year follow-up. We identified 3 distinct latent classes of mental health comorbidities for veterans with PTSD and T2D, i.e., low comorbidity (72%), depression and anxiety (20.9%), and high comorbidity (7.1%). These classes align with prior studies on latent classes of psychiatric comorbidities in individuals with PTSD [24,25]. In fact, the distribution of classes is similar to a nationally representative U.S. sample of individuals with PTSD [24], where the majority were in the low comorbidity class (62.1%), followed by the mood and anxiety class (23.7%), and the high comorbidity class (defined as mood, anxiety and substance dependence) (14.1%).

Furthermore, we found that, after controlling for confounders, veterans with PTSD and T2D in the depression + anxiety and high co-morbidity classes compared to the low comorbidity class had a significantly increased risk for initiating insulin therapy, experiencing macrovascular complications, and all-cause mortality. The depression + anxiety class, compared to the low comorbidity class, also had a marginally increased risk for poor glycemic control and microvascular complications, while the high comorbidity class did not. These findings are surprising as the high comorbidity group does not appear to be the most severely impacted. In fact, the depresssion + anxiety class consistently had the worst outcomes. This unexpected finding for the high comorbidity class may be explained by more surveillance and increased interactions with health care providers that served as a protective factor; specifically, these veterans likely receive more intensive, integrated case management and frequent clinical touchpoints within the VHA system, which may lead to better incidental monitoring of T2D.

The fact that the depression + anxiety class had the worst outcomes suggests that these outcomes may be driven by synergistic dysregulation of the hypothalamic–pituitary–adrenal (HPA) axis, which leads to and exacerbates insulin resistance and hyperglycemia via lipogenesis, adiposity, and inflammation [26]. The co-occurrence of depression and anxiety with PTSD may constantly activate the HPA Axis and accelerate these metabolic pathways more severely than in other comorbidity profiles.

Moreover, we found the association between comorbidity classes and T2D outcomes varied by age, gender, and race. While we generally found that the risk for T2D complications increased with age, we found that the association between the depression + anxiety and high co-morbidity classes and mortality risk was highest in younger veterans. This suggests that the presence of multiple psychiatric conditions may lead to “accelerated aging” or an earlier onset of metabolic burden that the body is less able to compensate for overtime. Regarding sex, males in the high comorbidity class showed a significantly greater risk for insulin initiation. Literature suggests that males are less likely to monitor their glucose and have different health-seeking behaviors compared to females [27]. Finally, the observation that White and Black veterans in the depression + anxiety class faced higher risks for poor glycemic control and microvascular complications is consistent with previous literature showing that racial minorities are more likely to have worse management of T2D and more T2D complications [28–30]. Though, notably, White veterans also showed this pattern which suggests that the depression + anxiety class may be driving worse outcomes. We found effect modification by race for some, but not all of the outcomes examined, with the association of high comorbidity and microvascular complications being higher and with poor glycemic control being lower in White and Black veterans compared to those in other racial groups. Prior studies have found less variation in mortality by race in the VHA as compared to private health care systems, and this is theorized to be partially due to VHA’s eligibility rules and access programs that reduce financial and other barriers to engaging with care. Further research is needed to better understand these observed sociodemographic differences. These sociodemographic factors are important to consider as they help identify patients with PTSD that are at greatest risk for microvascular and macrovascular complications of T2D.

### 4.1. Clinical & public health implications

Veterans with PTSD and comorbid psychiatric conditions, particularly depression and anxiety, are at higher risk for T2D complications. These findings emphasize the urgent need for evidence-based screening and treatment strategies tailored to this complex patient population. As such, further research is needed and should prioritize the PTSD and mood disorder comorbidity profile as it appears to drive the poorest metabolic outcomes. Such screening can then inform integrated care approaches that simultaneously address T2D and psychiatric symptoms, such as the Primary Care Mental Health Integration (PCMHI) clinics within the VA.

Furthermore, the counterintuitive finding that the high-comorbidity group were more likely to have controlled HbA1c may reflect the protective benefit of frequent healthcare contact and intensive case management [31,32]. Consequently, providers should consider closer monitoring by increasing appointment frequency or implementing intensive care coordination for patients presenting specifically with PTSD, depression and anxiety, to mimic this surveillance benefit.

From a public health perspective, the substantial mortality risk associated with the PTSD, depression and anxiety class of veterans with T2D highlights an urgent need to increase co-located physical and mental health services, like those provided by Primary Care Mental Health Integration clinics (PCMHI) within the VHA. These integrated services would allow for co-treatment and co-management of glycemic control and psychiatric symptoms, potentially reducing the long-term burden of T2D complications.

### 4.2. Strengths, limitations & future directions

Employing person-centered approaches like Latent Class Analysis (LCA) can better capture the real-world heterogeneity of psychiatric comorbidities. This statistical method identifies unobserved subgroups or “latent classes” of individuals based on their shared patterns of characteristics. While others have computed psychiatric comorbidity latent classes, to our knowledge this is the first study of comorbidity profiles and T2D outcomes and the first to utilize a patient-centered approach in examining T2D outcomes in patients with PTSD. Such an approach closely mirrors real-world clinical presentations and therefore may be more informative than traditional, siloed approaches for clinical practice in treating patients with co-morbid PTSD and T2D; this is because traditional approaches either only consider treating one condition at a time or separate out mental health from physical health thus precluding integrative treatment approaches. Next, this study utilizes a large cohort with an extensive follow-up to allow for robust measurement of long-term T2D complications.

The study also has limitations that warrant consideration. First, these findings may not generalize to non-veteran populations. Therefore, further research is needed to determine whether findings are similar in non-VHA health care settings. Eligibility required the last HbA1c value in the year prior to index < 7.5%. By sampling on controlled T2D, we may have under-estimated the magnitude of the relationship between latent classes and T2D outcomes to be followed by poor glycemic control for microvascular complications given good baseline control. Future research should employ prospective cohort designs to evaluate how concurrent changes in symptoms across multiple psychiatric conditions influence T2D outcomes over time. Future studies should also examine potential mechanisms in these associations. Given the lack of research examining latent comorbidity profiles and their association with physical health outcomes, future research should also examine these PTSD comorbidity profiles in the context of other physical health outcomes. We did not compute cause specific mortality and future research is needed to determine if variation in comorbidity patterns are followed by unique or common causes of death. Finally, unmeasured confounding is always a potential limitation and risk of misclassification of exposure or outcomes could bias measures of association.

### 4.3. Conclusion

Veterans with PTSD and comorbid psychiatric conditions, particularly depression and anxiety, are at higher risk for T2D complications, including poor glycemic control, insulin initiation, microvascular complications, and all-cause mortality. The findings underscore the importance of screening for additional psychiatric conditions in T2D patients with PTSD and to consider these conditions in tandem along sociodemographic factors to provide person-centered care and improve management of T2D that is comorbid with PTSD and other psychiatric disorders.

## Supplementary Material

1

## Figures and Tables

**Fig. 1.**
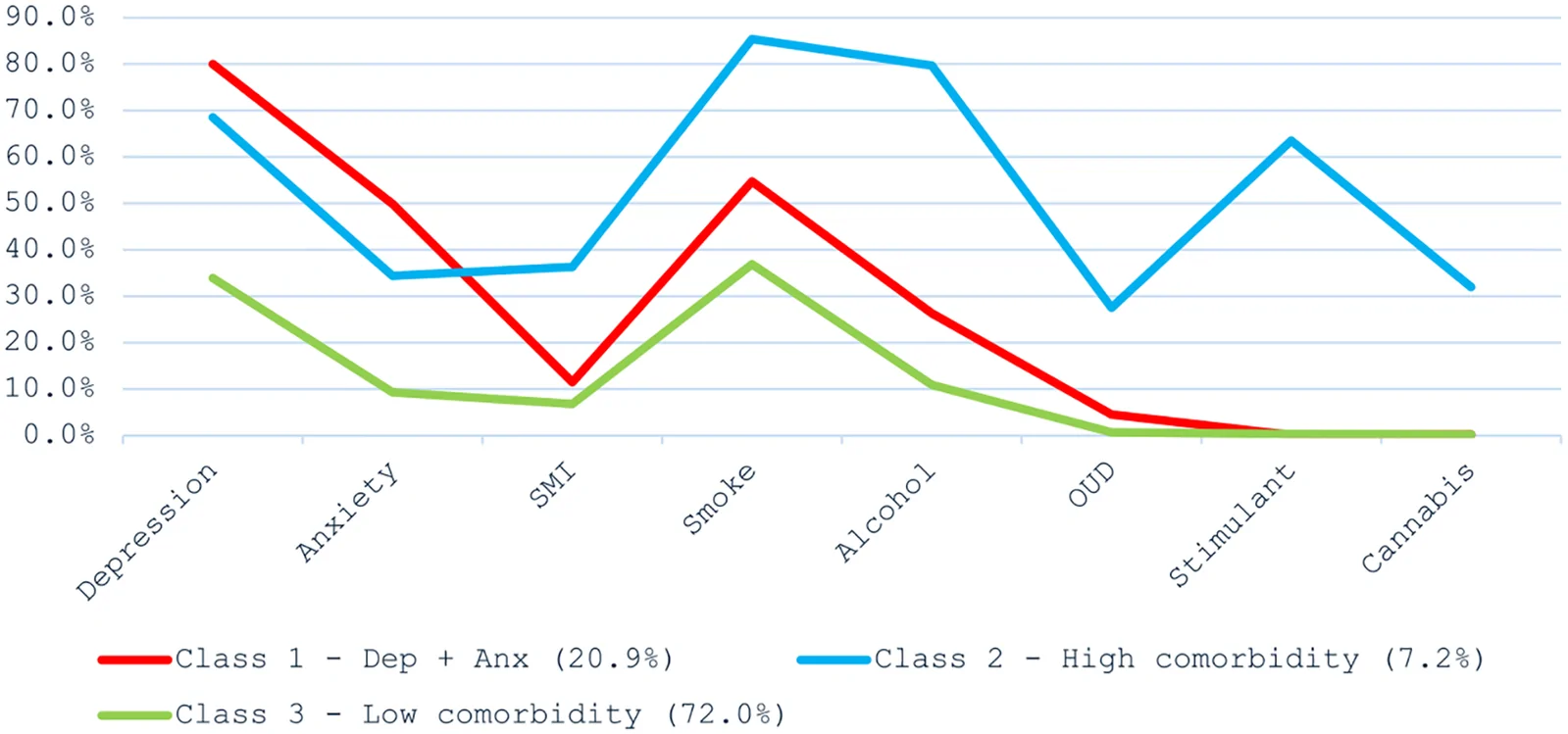
Latent Class Analyses: PTSD comorbidity profiles.

**Table 1:** Baseline characteristics and outcomes for comorbid PTSD-T2D veterans (n = 152,171).

	n(%) or mean(±sd)
**Demographics**	
Age	
18–39	7731 (5.1)
40–49	18,316 (12.0)
50–59	28,146 (18.5)
≥60	97,978 (64.4)
Male gender	138,398 (90.9)
Race	
Black	41,115 (27.0)
Other	5970 (3.9)
White	105,086 (69.1)
Married	93,014 (61.1)
Region	
Northeast	19,095 (12.6)
Midwest	25,313 (16.6)
South	76,497 (50.3)
West	31,266 (20.6)
VA only insurance	40,368 (26.5)
High healthcare utilization	37,835 (24.9)
**Type 2 diabetes factors**	
HbA1c, mean(±sd)	6.3 (±0.6)
*Diabetes drugs (sustained use)*	
Metformin	59,026 (38.8)
Sulfonylurea	18,303 (12.0)
Other diabetes drug	3216 (2.1)
**Physical comorbidities**	
Obesity	95,598 (62.8)
Hyperlipidemia	120,210 (79.0)
Atrial Fibrillation	10,331 (6.8)
Angina	2400 (1.6)
Congestive heart failure	10,917 (7.2)
Hypertension	121,222 (79.7)
Left ventricular hypertrophy	5214 (3.4)
MI	3406 (2.2)
Peripheral vascular disease	8022 (5.3)
Stroke	8412 (5.5)
**Psychiatric Treatment**	
Adequate PTSD treatment	9267 (6.1)
ADM ≥ 12 weeks	99,502 (65.4)
Atypical antipsychotic	27,297 (17.9)
**Psychiatric comorbidities**	
Depression	76,551 (50.3)
Anxiety	35,466 (23.3)
SMI	16,324 (10.7)
Smoke	70,515 (46.3)
Alcohol abuse/dependence	32,504 (21.4)
Opioid abuse/dependence	6328 (4.2)
Stimulant abuse/dependence	8824 (5.8)
Cannabis abuse/dependence	4585 (3.0)

**Table 2:** Baseline characteristics for comorbid PTSD-T2D veterans, by PTSD-Comorbidity Class (n = 152,171).

	Dep + Anx (n = 31813)	high comorbidity (n = 10741)	Low comorbidity (n = 109617)	p-value	Max Unwt SMD%	Max wt SMD%
Age				0<.0001		
18–39	1945 (6.1)	650 (6.1)	5136 (4.7)		6.2	0.00
40–49	4500 (14.2)	1374 (12.8)	12,442 (11.4)		8.4	0.00
50–59	7054 (22.2)	3731 (34.7)	17,361 (15.8)		44.9	0.00
≥60	18,314 (57.6)	4986 (46.4)	74,678 (68.1)		44.6	0.00
Male gender	27,482 (86.4)	9832 (91.5)	101,084 (92.2)	0<.0001	19.6	0.00
Race				0<.0001		
Black	6803 (21.4)	5264 (49.0)	29,048 (26.5)		61.1	0.00
Other	1136 (3.6)	303 (2.8)	4531 (4.1)		7.1	0.00
White	23,874 (75.0)	5174 (48.2)	76,038 (69.4)		57.8	0.00
Married	18,207 (57.2)	2991 (27.9)	71,816 (65.5)	0<.0001	79.6	0.00
Region				0<.0001		
Northeast	3941 (12.4)	1808 (16.8)	13,346 (12.2)		13.5	0.00
Midwest	6311 (19.8)	1919 (17.9)	17,083 (15.6)		11.1	0.00
South	15,722 (49.4)	4839 (45.1)	55,936 (51.0)		12.0	0.00
West	5839 (18.4)	2175 (20.2)	23,252 (21.2)		7.2	0.00
VA only insurance	9057 (28.5)	5213 (48.5)	26,098 (23.8)	0<.0001	53.8	0.00
High HCU	10,980 (34.5)	6249 (58.2)	20,606 (18.8)	0<.0001	86.5	0.00
HbA1c, mean(±sd)	6.2 (±0.6)	6.2 (±0.6)	6.3 (±0.6)	0<.0001	23.5	0.00
Metformin	11,252 (35.4)	3651 (34.0)	44,123 (40.3)	0<.0001	13.0	0.00
Sulfonylurea	3038 (9.6)	1033 (9.6)	14,232 (13.0)	0<.0001	11.1	0.00
Other diab drug	540 (1.7)	94 (0.9)	2582 (2.4)	0<.0001	11.7	0.00
Adeq PTSD treatment	2258 (7.1)	1040 (9.7)	5969 (5.5)	0<.0001	16.2	0.00
Obesity	19,852 (62.4)	5607 (52.2)	70,139 (64.0)	0<.0001	24.1	0.00
Hyperlipidemia	25,418 (79.9)	7559 (70.4)	87,233 (79.6)	0<.0001	22.6	0.00
Atrial Fibrillation	2650 (8.3)	640 (6.0)	7041 (6.4)	0<.0001	9.4	0.00
Angina	725 (2.3)	207 (1.9)	1468 (1.3)	0<.0001	7.0	0.00
Congestive heart failure	3121 (9.8)	1017 (9.5)	6779 (6.2)	0<.0001	13.0	0.00
Hypertension	25,624 (80.6)	8572 (79.8)	87,026 (79.4)	0<.0001	2.9	0.00
Left ventricular hypert	1614 (5.1)	386 (3.6)	3214 (2.9)	0<.0001	11.1	0.00
MI	1076 (3.4)	337 (3.1)	1993 (1.8)	0<.0001	9.5	0.00
Peripheral vascular dis	2274 (7.2)	601 (5.6)	5147 (4.7)	0<.0001	10.5	0.00
Stroke	2276 (7.2)	649 (6.0)	5487 (5.0)	0<.0001	9.0	0.00
ADM ≥ 12 weeks	25,335 (79.6)	7695 (71.6)	66,472 (60.6)	0<.0001	42.3	0.00
Atypical antipsychotic	8355 (26.3)	4431 (41.3)	14,511 (13.2)	0<.0001	65.4	0.00

**Table 3:** Outcomes: cumulative incidence percent and incidence rate, by PTSD-Comorbidity class (n = 152,171).

Outcome	Dep + Anx (n = 31813)	High comorbidity (n = 10741)	Low comorbid (n = 109617)	p-value
*Starting insulin*				
#events (%)	3877 (12.2)	1510 (14.1)	12,058 (11.0)	0<.0001
Incidence rate per 1,000PY	23.0/1000PY	26.6/1000PY	19.6/1000PY	0<.0001
*Poor glycemic control*				
#events (%)	11,580 (36.4)	3648 (34.0)	43,956 (40.1)	0<.0001
Incidence rate per 1,000PY	93.5/1000PY	85.6/1000PY	99.9/1000PY	0<.0001
*Any microvascular complication*				
#events (%)	15,660 (49.2)	5038 (46.9)	56,646 (51.7)	0<.0001
Incidence rate per 1,000PY	130.2/1000PY	119.0/1000PY	134.1/1000PY	0<.0001
*All-cause Mortality*				
#events (%)	4902 (15.4)	2085 (19.4)	13,559 (12.4)	0<.0001
Incidence rate per 1,000PY	26.2/1000PY	32.6/1000PY	20.2/1000PY	0<.0001

**Table 4:** Outcomes: Competing risk survival and Cox proportional hazard models, before and after entropy balancing. (n = 152,171).

Outcome	Dep + Anx HR (95% CI)	High comorbidity HR (95% CI)	Low comorbid HR (95% CI)
*Starting insulin* ^a^			
Unweighted	1.14 (1.10–1.18)	1.30 (1.24–1.38)	1.00
Weighted	1.14 (1.10–1.18)	1.29 (1.22–1.36)	1.00
*Poor glycemic control* ^a^			
Unweighted	0.91 (0.89–0.93)	0.83 (0.80–0.86)	1.00
Weighted	1.04 (1.02–1.06)	0.99 (0.96–1.03)	1.00
*Any microvascular complication* ^a^			
Unweighted	0.95 (0.93–0.97)	0.86 (0.84–0.89)	1.00
Weighted	1.04 (1.02–1.06)	0.99 (0.96–1.02)	1.00
*Mortality* ^b^			
Unweighted	1.32 (1.28–1.36)	1.63 (1.56–1.71)	1.00
Weighted	1.18 (1.14–1.23)	1.39 (1.28–1.50)	1.00

a Competing risk survival models.

b Cox proportional hazard model.

## Data Availability

Data will be made available after completion of funding period and via direct request to the corresponding author.

## Appendix A. Supplementary data

Supplementary data to this article can be found online at https://doi.org/10.1016/j.diabres.2026.113257.
