# Supplementary material for: Type 2 diabetes outcomes in patients with Posttraumatic stress disorder and psychiatric comorbidity
Source: Diabetes Res Clin Pract. Author manuscript; Available in PMC 2026 Nov 16. (PMC13771907; doi:10.1016/j.diabres.2026.113257)
Supplement: Supplementary file 1 [file NIHMS2221935-supplement-1.docx]

**APPENDIX**

| **Appendix A: e-Table 1.** | |
| --- | --- |
| **Variable** | **Definition** |
| Index/baseline | First time PTSD diagnostic algorithm met where: a) comorbid T2D in 1-year prior; b) no T1DM or insulin fills in 2-years prior; and c) no microvascular complications in 2-years prior |
| Follow-up time | Months from index to study end date (incident outcome or censoring).  Censoring for each outcome:  Time to insulin start – death or last visit  Time to microvascular complication – death or last visit  Time to poor glycemic control – death or last HbA1c measurement  All-cause mortality – 9/30/22 |
| T2DM | ICD-9 code: 250.x0, 250.x2;  ICD-10 code: E11.x |
| PTSD | ICD-9 code: 309.81;  ICD-10 code: F43.1x  - 2 outpatient occurrences (on different days) in same 12-month period or 1 inpatient occurrence |
| T1DM | ICD-9 code: 250.x1, 250.x3;  ICD-10 code: E10.x |
| **Outcomes** | |
| Starting insulin | First insulin fill in follow-up |
| Poor glycemic control | First HbA1c in follow-up ≥7.5% |
| Any microvascular complication | Composite from list below: **Microvascular complication** \| **ICD9** \| **ICD10**; Diabetic retinopathy \| 250.50, 250.52, 362.0x, 366.41 \| E11.3x, E13.3x, E14.3x, H28.0, H36.0; Diabetic neuropathy \| 250.60, 250.62, 357.2 \| E11.4x, E13.4x, E14.4x, G63.2, G59.0; Diabetic nephropathy \| 250.40, 250.42, 585.x \| E11.2x, E13.2x, E14.2x, N08.3, N18.x |
| Mortality | All-cause mortality from VHA vital status file |
| Secondary outcome: CVD (macrovascular complication) | Composite of ischemic heart disease, cardiomyopathy, arrhythmias, AFIB  ICD-9 code: 410.x-414.x , 425.x, 427.x  ICD-10 code: I20.x-I25.x, I42.x, I43.x, I47.x, I48.x, I49.x |
| Secondary outcome: PVD (microvascular complication) | ICD-9 code: 440.2x, 440.3x, 440.4, 440.9;  ICD-10 code: I70.x, I73.1, I73.9 |
| **Exposure** | |
| Comorbidity class | Low comorbidity vs. depression+anxiety vs. high comorbidity, defined by LCA using 8 indicators listed below, that were measured in the 2-years prior to index. |
| Depression | ICD-9 code: 296.2x, 296.3x, 311;  ICD-10 code: F32.0-F32.5, F32.9, F33.0-F33.3, F33.4x, F33.9  - 2 outpatient occurrences (on different days) in same 12-month period or 1 inpatient occurrence |
| Anxiety | ICD-9 code: 300.00, 300.01, 300.02, 300.23, 300.3;  ICD-10 code: F40.1x, F41.0, F41.1, F41.9, F42  - Composite of panic disorder, anxiety disorder not otherwise specified, social phobia, and generalized anxiety disorder, OCD  - 2 outpatient occurrences (on different days) in same 12-month period or 1 inpatient occurrence |
| Severe mental illness | ICD-9 code: 295.x, 296.0x, 296.1x, 296.4x-296.8x;  ICD-10 code: F20.x, F25.x, F30.x, F31.x  Composite of schizophrenia or bipolar disorder |
| Smoking/nicotine dependence | ICD-9 code: V15.82, 305.1;  ICD-10 code: Z87.891, Z72.0, F17.20x, F17.21x  Present in health factor data as current smoker |
| Alcohol abuse/dependence | ICD-9 code: 303.9x, 305.0x;  ICD-10 code: F10.x |
| Opioid abuse/dependence | ICD-9 code: 304.0x, 304.7x, 305.5x;  ICD-10 code: F11.x |
| Stimulant abuse/dependence | ICD-9 code: 304.2x, 304.4x, 305.6x, 305.7x;  ICD-10 code:, F14.x, F15.x  -Composite of cocaine or amphetamine/psychostimulant |
| Cannabis abuse/dependence | ICD-9 code: 304.3x, 305.2x;  ICD-10 code: F12.x |
| **Comorbidity covariates – measured in 2-years prior to index. At least one occurrence/diagnostic code unless otherwise specified.** | |
| Adequate PTSD treatment | At least 9 unique clinic stops (clinic stop codes: 516, 540, 541, 561, 562) for PTSD psychotherapy in any 15-week period. |
| Adequate ADM treatment | At least 12 weeks of continuous use. Continuous use is defined as no gaps > 30 days in fills. All SSRI, SNRI, MAOI, TCA, and other types. |
| Atypical antipsychotic use | Sustained use = is at least 2 fills in any 6-month period. Atypical antipsychotics: aripiprazole, asenapine, brexipiprazole, cariprazine, clozapine, iloperidone, lurasidone, olanzapine, paliperidone, pimavanserin, quetiapine, risperidone, ziprasidone |
| Obesity | Last BMI on/before index is ≥ 30 |
| Hyperlipidemia | ICD-9 code: 272.0 – 272.4;  ICD-10 code: E78.0x, E78.1, E78.2, E78.3, E78.4x, E78.5, E78.8x, E78.9 |
| Atrial Fibrillation | ICD-9 code: 427.3x;  ICD-10 code: I48.x |
| Angina | ICD-9 code: 411.81, 411.89, 413.0;  ICD-10 code: I20.0, I24.8, I24.9, I25.110, I25.700, I25.710, I25.720, I25.730, I25.750, I25.760, I25.790 |
| Congestive heart failure | ICD-9 code: 398.91, 402.01, 402.11, 402.91, 404.01, 404.03, 404.11, 404.13, 404.91, 404.93, 428.x;  ICD-10 code: I09.81, I11.0, I13.0, I13.2, I50x |
| Hypertension | ICD-9 code: 401.x – 405.x;  ICD-10 code: I10, I11.x, I12.x, I13.x, I15.x, I16.x |
| Left ventricular hypertrophy | ICD-9 code: 425.1, 429.3;  ICD-10 code: I42.1, I42.2, I51.7 |
| MI | ICD-9 code: 410.x, 411.0;  ICD-10 code: I21.x, I22.x, I23.x |
| Peripheral vascular disease | ICD-9 code: 440.2x, 440.3x, 440.4, 440.9;  ICD-10 code: I70.x, I73.1, I73.9 |
| Stroke | ICD-9 code: 430, 431, 433.x, 434.x;  ICD-10 code: I60.x, I61.x, I63.x |
| Diabetes drugs | Sustained use = at least 2 fills in any 6-month period. Separate variables for:  Metformin; sulfonylurea; other drug (i.e., GLP-1 receptor antagonists; SGLT-2 inhibitors; DPP-4 inhibitors; TZD’s) |
| **Demographic and other information** | |
| Age at index | 18-39, 40-49, 50-59, ≥60 (60-80) |
| Race | Black, White, Other |
| Sex | Male, female |
| Marital status | Married vs. other (on/closest to index date). |
| VA only health insurance vs. VA+private/Other | - Insurance status variable indicating any access to non-VA health insurance. Coverage defined by most frequently occurring coverage in 2-years prior to index date |
| Region | Home state geographic region at index. VHA provides home state, which was further categorized based on census region: northeast, Midwest (north central), south, west. |
| High health services utilization | - Controls for detection bias related to more healthcare encounters.  - Average number of outpatient clinic visits per month, calculated as total visits divided by number of months. Total visits is total number of outpatient visits in the two years prior to index and number of months for denominator is 24. The distribution average number of visits per month per patient is then dichotomized into high utilizer, >75^th^ percentile vs low utilizer, ≤75^th^ percentile. |

| **Appendix e-Table 2. Latent Class Analyses model fit statistics. The 3-Class solution best fit the data** | | | | | | | | |
| --- | --- | --- | --- | --- | --- | --- | --- | --- |
| Classes | #par | LL | AIC | BIC | aBIC | Entropy | aLMR, p-value | Class prob |
| 1 | 8 | -504531.2 | 1009078.3 | 1009157.8 | 1009132.4 | na | na | 100% |
| 2 | 17 | -477706.0 | 955445.9 | 955614.8 | 955560.8 | 0.82 | <.0001 | 10.5%; 89.5% |
| **3** | **26** | **-474062.4** | **948176.8** | **948435.0** | **948352.4** | **0.58** | **<.0001** | **20.9%; 7.1%; 72.0%** |
| 4 | 35 | -472808.2 | 945686.3 | 946033.9 | 945922.7 | 0.69 | <.0001 | 70.2%; 4.7%; 11.3%; 13.7% |
| 5 | 44 | -472202.9 | 944493.9 | 944930.9 | 944791.1 | 0.74 | <.0001 | 5.1%; 4.7%; 37.8%; 9.5%; 42.9% |

| **Appendix e-Table 3. Item response probabilities (95% confidence intervals) given class membership (n=152,171)** | | | |
| --- | --- | --- | --- |
| **Variable** | **Class 1 - Dep + Anx (20.9)** | **Class 2 - High Comorbidity (7.1)** | **Class 3 - Low Comorbidity (72.0)** |
|  | (95 CI) | (95 CI) | (95 CI) |
| Depression | 80.0 (78.1-81.9) | 68.5 (67.5-69.5) | 33.9 (32.9-34.9) |
| Anxiety | 49.9 (47.9-51.9) | 34.4 (33.5-35.4) | 9.3 (8.6-10.1) |
| SMI | 11.5 (10.9-12.1) | 36.3 (35.3-37.3) | 6.8 (6.5-7.0) |
| Smoke | 54.7 (53.5-56.0) | 85.4 (84.6-86.1) | 36.9 (36.3-37.5) |
| Alcohol | 26.3 (25.2-27.3) | 79.7 (78.7-80.6) | 10.9 (10.4-11.3) |
| OUD | 4.5 (4.1-4.9) | 27.5 (26.6-28.5) | 0.7 (0.6-0.8) |
| Stimulant | 0.1 (-0.1-0.3) | 63.5 (61.2-65.8) | 0.3 (0.2-0.4) |
| Cannabis | 0.2 (0.01-0.03) | 32.0 (30.6-33.3) | 0.2 (0.1-0.3) |

| **Appendix e-Table 4. Average latent class posterior probabilities for most likely latent class membership (row) by latent class (column) (n=152,171)** | | | |
| --- | --- | --- | --- |
|  | **Class 1 - Dep + Anx** | **Class 2 - High Comorbidity** | **Class 3 - Low Comorbidity** |
| Class 1 | 74.0 | 6.8 | 19.3 |
| Class 2 | 3.2 | 93.8 | 3.0 |
| Class 3 | 18.5 | 1.0 | 80.5 |

| **Appendix, e-Table 5.** Outcomes: Competing risk survival and Cox proportional hazard models. After entropy balancing. Stratified by age | | | |
| --- | --- | --- | --- |
| Outcome (weighted models) | Dep + Anx  HR (95% CI) | High comorbidity HR (95% CI) | Low comorbidity  HR (95% CI) |
| *Starting insulin^a^* |  |  |  |
| Age 18-39 | 1.07 (0.94-1.22) | 0.92 (0.74-1.15) | 1.00 |
| Age 40-49 | 1.13 (1.03-1.23) | 1.17 (1.02-1.33) | 1.00 |
| Age 50-59 | 1.10 (1.02-1.19) | 1.37 (1.22-1.54) | 1.00 |
| Age 60-80 | 1.15 (1.10-1.21) | 1.28 (1.19-1.38) | 1.00 |
| *p-value age*class* | *p=.047* | |  |
|  |  |  |  |
| *Poor glycemic control^a^* |  |  |  |
| Age 18-39 | 0.98 (0.91-1.07) | 0.95 (0.82-1.09) | 1.00 |
| Age 40-49 | 1.00 (0.95-1.06) | 0.88 (0.81-0.96) | 1.00 |
| Age 50-59 | 1.00 (0.96-1.05) | 1.01 (0.94-1.08) | 1.00 |
| Age 60-80 | 1.06 (1.03-1.09) | 1.02 (0.97-1.07) | 1.00 |
| *p-value age*class* | *p=.011* | |  |
|  |  |  |  |
| *Any microvascular complication^a^* |  |  |  |
| Age 18-39 | 1.05 (0.93-1.18) | 0.92 (0.74-1.14) | 1.00 |
| Age 40-49 | 1.11 (1.04-1.18) | 1.14 (1.03-1.26) | 1.00 |
| Age 50-59 | 1.07 (1.03-1.12) | 1.05 (0.97-1.12) | 1.00 |
| Age 60-80 | 1.03 (1.01-1.05) | 0.95 (0.92-0.99) | 1.00 |
| *p-value age*class* | *p=.001* | |  |
|  |  |  |  |
| *Mortality^b^* |  |  |  |
| Age 18-39 | 1.59 (1.13-2.25) | 3.51 (2.19-5.65) | 1.00 |
| Age 40-49 | 1.48 (1.23-1.79) | 2.11 (1.58-2.84) | 1.00 |
| Age 50-59 | 1.51 (1.35-1.69) | 1.88 (1.60-2.20) | 1.00 |
| Age 60-80 | 1.16 (1.11-1.20) | 1.32 (1.19-1.46) | 1.00 |
| *p-value age*class* | *p=.<.0001* | |  |
| ^a^ Competing risk survival models  ^b^ Cox proportional hazard model | | | |

| **Appendix, e-Table 6.** Outcomes: Competing risk survival and Cox proportional hazard models. After entropy balancing. Stratified by sex | | | |
| --- | --- | --- | --- |
| Outcome (weighted models) | Dep + Anx  HR (95% CI) | High comorbidity HR (95% CI) | Low comorbidity  HR (95% CI) |
| *Starting insulin^a^* |  |  |  |
| Female | 1.20 (1.07-1.35) | 0.87 (0.71-1.09) | 1.00 |
| Male | 1.13 (1.09-1.17) | 1.32 (1.25-1.39) | 1.00 |
| *p-value sex*class* | *p=.0002* | |  |
|  |  |  |  |
| *Poor glycemic control^a^* |  |  |  |
| Female | 1.10 (1.02-1.18) | 0.95 (0.85-1.07) | 1.00 |
| Male | 1.03 (1.01-1.05) | 1.00 (0.96-1.03) | 1.00 |
| *p-value sex*class* | *p=.198* | |  |
|  |  |  |  |
| *Any microvascular complication^a^* |  |  |  |
| Female | 1.07 (0.99-1.14) | 1.06 (0.95-1.18) | 1.00 |
| Male | 1.04 (1.02-1.06) | 0.98 (0.95-1.01) | 1.00 |
| *p-value age*class* | *p=.303* | |  |
|  |  |  |  |
| *Mortality^b^* |  |  |  |
| Female | 1.32 (1.12-1.57) | 1.94 (1.38-2.73) | 1.00 |
| Male | 1.18 (1.14-1.22) | 1.37 (1.26-1.49) | 1.00 |
| *p-value sex*class* | *p=.096* | |  |
| ^a^ Competing risk survival models  ^b^ Cox proportional hazard model | | | |

| **Appendix, e-Table 7.** Outcomes: Competing risk survival and Cox proportional hazard models. After entropy balancing. Stratified by race | | | |
| --- | --- | --- | --- |
| Outcome (weighted models) | Dep + Anx  HR (95% CI) | High comorbidity HR (95% CI) | Low comorbidity  HR (95% CI) |
| *Starting insulin^a^* |  |  |  |
| Black | 1.17 (1.08-1.26) | 1.39 (1.25-1.54) | 1.00 |
| White | 1.14 (1.09-1.18) | 1.25 (1.17-1.33) | 1.00 |
| Other | 0.97 (0.80-1.18) | 1.30 (0.98-1.69) | 1.00 |
| *p-value age*class* | *p=.213* | |  |
|  |  |  |  |
| *Poor glycemic control^a^* |  |  |  |
| Black | 1.06 (1.02-1.10) | 1.06 (0.99-1.13) | 1.00 |
| White | 1.03 (1.01-1.06) | 0.96 (0.92-0.99) | 1.00 |
| Other | 0.99 (0.90-1.10) | 1.21 (1.05-1.41) | 1.00 |
| *p-value age*class* | *p=.002* | |  |
|  |  |  |  |
| *Any microvascular complication^a^* |  |  |  |
| Black | 1.04 (1.01-1.08) | 1.04 (0.99-1.10) | 1.00 |
| White | 1.04 (1.02-1.06) | 0.97 (0.94-1.01) | 1.00 |
| Other | 1.03 (0.94-1.14) | 0.69 (0.58-0.82) | 1.00 |
| *p-value age*class* | *p=.0003* | |  |
|  |  |  |  |
| *Mortality^b^* |  |  |  |
| Black | 1.16 (1.06-1.27) | 1.32 (1.17-1.48) | 1.00 |
| White | 1.18 (1.14-1.23) | 1.42 (1.28-1.57) | 1.00 |
| Other | 1.25 (1.01-1.55) | 1.93 (1.15-3.23) | 1.00 |
| *p-value age*class* | *p=.620* | |  |
| ^a^ Competing risk survival models  ^b^ Cox proportional hazard model | | | |

| **Appendix e-Table 8.** Secondary Outcomes: Macrovascular outcomes, cumulative incidence percent, and incidence rate, by PTSD-Comorbidity class (n=100,610) | | | | |
| --- | --- | --- | --- | --- |
| Outcome | Dep + Anx  (n=19459) | High comorbidity  (n=7123) | Low comorbid  (n=74028) | p-value |
| *CVD* |  |  |  |  |
| #events (%) | 6687 (34.4) | 2657 (37.3) | 24623 (33.3) | <.0001 |
| Incidence rate per 1,000PY | 75.0/1000PY | 81.4/1000PY | 69.6/1000PY | <.0001 |
|  |  |  |  |  |
| *PVD* |  |  |  |  |
| #events (%) | 2547 (13.1) | 918 (12.9) | 9727 (13.1) | .830 |
| Incidence rate per 1,000PY | 24.2/1000PY | 23.4/1000PY | 23.6/1000PY | .547 |
|  |  |  |  |  |
|  | | | | |

| **Appendix e-Table 9**. Macrovascular Outcomes: Competing risk survival models, before and after entropy balancing. (n=100,610) | | | |
| --- | --- | --- | --- |
| Outcome | Dep + Anx  HR (95% CI) | High Comorbidity  HR (95% CI) | Low comorbid  HR (95% CI) |
| *CVD* |  |  |  |
| Unweighted | 1.06 (1.04-1.09) | 1.13 (1.09-1.18) | 1.00 |
| Weighted | 1.11 (1.08-1.14) | 1.13 (1.09-1.18) | 1.00 |
|  |  |  |  |
| *PVD* |  |  |  |
| Unweighted | 1.01 (0.97-1.05) | 0.96 (0.89-1.03) | 1.00 |
| Weighted | 1.10 (1.05-1.14) | 1.11 (1.04-1.18) | 1.00 |

| **Appendix e-Table 10. Example MPlus and R-studio syntax** | |
| --- | --- |
|  |  |
| **R-studio – WeightIt for entropy balancing and testing balance** | (w1<- weightit(classcat~age1+age2+age3+age4+malegender+blackrace+otherrace+  whiterace+married+ne+nc+so+we+vaonlyinsurance+high25util+a1cvalue+  adeqtxt+adm84days+ap+obese+hyperlipid+afib+angina+chf+htn+lvhypertroph+mi+pvd+stroke+metformin+su+otherdiabdrug, data=edata2,method="ebal", estimand='ATE', moments=2))  bal.tab(w1,un=TRUE,binary='std',continuous='std') |
|  |  |
| **MPlus – example latent class analysis syntax** | title: Final 3 class solution with CI  data:  file is lca1_novar.csv;  variable:  names=id dep anx smi smoke alcdep opdep stimdep candep;  usevariables=dep anx smi smoke alcdep opdep stimdep candep;  categorical=dep anx smi smoke alcdep opdep stimdep candep;  IDVARIABLE=id;  classes=c(3);  analysis:  type=mixture;  output:  tech11 tech14;  cinterval;  savedata:  file is lca3_final.txt;  save is cprob;  format is free; |

Appendix e-Figure 1 - Screen plot of aBIC vs. number of classes

Sample-size adjusted BIC (aBIC)

| Number of classes | aBIC |
| --- | --- |
| 1 | 1009132 |
| 2 | 955561 |
| 3 | 948352 |
| 4 | 945923 |
| 5 | 944791 |

**Appendix e-Figure 2. Cumulative incidence function for time to insulin initiation, after entropy balance weighting**

**Appendix e-Figure 3. Cumulative incidence function for time to poor glycemic control, after entropy balance weighting**

**Appendix e-Figure 4. Cumulative incidence function for time to any microvascular complications, after entropy balance weighting**
